# The Use of Novel Drugs Can Effectively Improve Response, Delay Relapse and Enhance Overall Survival in Multiple Myeloma Patients with Renal Impairment

**DOI:** 10.1371/journal.pone.0101819

**Published:** 2014-07-08

**Authors:** Katarina Uttervall, Adil D. Duru, Johan Lund, Johan Liwing, Gösta Gahrton, Erik Holmberg, Johan Aschan, Evren Alici, Hareth Nahi

**Affiliations:** 1 Department of Medicine, Karolinska Institutet Huddinge, Stockholm, Sweden; 2 Hematology Center, Karolinska University Hospital Huddinge, Stockholm, Sweden; 3 Department of Oncology, Institute of Clinical Sciences, Sahlgrenska Academy at University of Gothenburg, Sweden; 4 Janssen-Cilag AB, Sollentuna, Sweden; Sapporo Medical University, Japan

## Abstract

**Background:**

Renal impairment is a common feature in multiple myeloma and is considered a poor prognostic factor.

**Aim:**

To determine the impact of novel drugs (i.e. bortezomib, lenalidomide and thalidomide) in the treatment of myeloma patients with renal impairment. The primary endpoint was overall survival and secondary endpoints were time to next treatment and response.

**Methods:**

The study population included all patients diagnosed with treatment-demanding multiple myeloma January 2000 to June 2011 at 15 Swedish hospitals. Renal impairment was defined as an estimated glomerular filtration rate under 60 mL/min/1.73 m^2^.

**Result:**

The study population consisted of 1538 patients, of which 680 had renal impairment at diagnosis. The median overall survival in patients with renal impairment was 33 months, which was significantly shorter than 52 months in patients with normal renal function (*P*<0.001). Novel agents in first line improved overall survival (median 60 months) in non-high-dose treated patients with renal impairment (n = 143) as compared to those treated with conventional cytotoxic drugs (n = 411) (median 27 months) (*P*<0.001). In the multivariate analysis up front treatment with bortezomib was an independent factor for better overall survival in non-high-dose treated renally impaired patients. High-dose treated renally impaired patients had significantly better median overall survival than non-high-dose ones (74 versus 26 months) and novel drugs did not significantly improve survival further in these patients. Patients with renal impairment had both a shorter median time to next treatment and a lower response rate than those with normal renal function. However, novel drugs and high dose treatment lead to a significantly longer time to next treatment and the use of novel agents significantly improved the response rate of these patients.

**Conclusion:**

High dose treatment and novel drugs, especially bortezomib, can effectively overcome the negative impact of renal impairment in patients with multiple myeloma.

## Introduction

Although the five-year survival for multiple myeloma (MM) patients has gradually increased over the last decades, especially with the use of high-dose chemotherapy and novel agents such as protease inhibitors and immunomodulatory drugs (IMiDs), MM is still considered incurable by conventional means. For the majority of patients the goal of therapy i.e. prolonged survival and preventing progression is related to the quality of response to initial therapy and depth of remissions reached [1], [2].

Renal impairment (RI) is a relatively common feature of MM both at diagnosis and in relapsed/refractory MM [3]. With the use of conventional chemotherapy, it has been shown in several studies that renal impairment at diagnosis correlates to inferior survival [4], [5], significant morbidity and increased early death rate.

The current gold standard for the treatment of patients less than 65 years of age is high-dose chemotherapy (HDT) followed by autologous stem cell transplantation [6]. However, the use of alkylators during induction treatment is associated with low response rate in patients with MM and RI [7]. Dexamethasone alone or in combination with cyclophosphamide is superior to melphalan containing regimens, because the latter are more dependent on renal elimination [7]. The addition of novel agents such as bortezomib has resulted in a substantial increase in the number of patients responding to therapy when compared to conventional therapy. Bortezomib is a proteasome inhibitor that promotes apoptosis, prevents DNA repair and inhibits proliferation [8] and it is effective in both newly diagnosed MM and relapsed/refractory MM [9]. It can be administered at the full-approved dose and schedule in patients with impaired renal function, because neither the pharmacokinetics nor the primary metabolic pathway of bortezomib are affected by renal impairment [10], [11]. Moreover, other novel agents such as the IMiDs lenalidomide and thalidomide increase the susceptibility of tumor cells to lymphocyte-mediated immunosurveillance, which leads to enhanced cytotoxicity towards previously tolerated tumor cells [12], [13]. However, lenalidomide is mainly excreted by the kidney [14] while thalidomide pharmacokinetics is not affected by renal function [15]. Currently there are limited data for the efficacy of IMiDs in patients with MM and RI.

The objective of this study is to understand the impact of RI on response to treatment, time to next treatment (TTNT) and most importantly survival in the era of novel agents (i.e. bortezomib, lenalidomide and thalidomide). In this retrospective study, we demonstrate that the use of novel drugs, especially bortezomib can effectively improve response, increase TTNT and enhance overall survival (OS) in multiple myeloma patients with renal impairment.

## Materials and Methods

### Study population

Consecutive patients diagnosed with MM between January 2000 and June 2011 at 7 university clinics, 5 regional centers and 3 local hospitals in Sweden were included. All patients were identified from the Swedish Cancer Registry, which covers 96.3% of all individuals with malignant disease [16]. Patients were excluded if they had non-treatment demanding disease (watch and wait), plasma cell leukemia or solitary plasmacytoma. All data were collected from the hospitals' electronic medical records and, in the case of death, from the national death registry. The study has received ethical approval from the Regional Ethical Vetting Board in Stockholm, Sweden. No informed consent was obtained because this was not required by the ethical committee. The patient information was anonymized and de-identified prior to analysis.

For each patient data regarding age, sex, type of myeloma and extent of bone disease as well as laboratory measurements of calcium, hemoglobin, β2-microglobulin, albumin and creatinine were collected at the time of diagnosis. Serum M-protein and urine M-protein values were collected at baseline and every significant change in the M-protein level was noted. In general, these proteins were measured on a monthly basis or at the start of every treatment cycle. For each treatment line the types of drugs given for MM were noted, with specific start and stop dates for each drug or drug combination. In the case of allogeneic stem cell transplantation the date was recorded and the patient was censored in all analysis after this date.

The estimated glomerular filtration rate (eGFR) was calculated using the Modification of Diet in Renal Disease (MDRD) formula: eGFR in mL/min/1.73 m^2^ = 186.3×(S-creatinine/88.4)^−1.154^×(age)^−0.203^ (×0.742 if female) [17]. Renal function was classified according to National Kidney Foundation Kidney Disease Outcomes Quality Initiative (KDOQI) chronic kidney disease classification [18] (Table 1). In this study RI was defined as eGFR <60 mL/min/1.73 m^2^.

**Table 1:** Classification of renal function according to National Kidney Foundation Kidney Disease Outcomes Quality Initiative.

Stage	Estimated glomerular filtration rate (mL/min/1.73 m^2^)
1	≥90
2	60–89
3	30–59
4	15–29
5	<15

**Figure 1:**
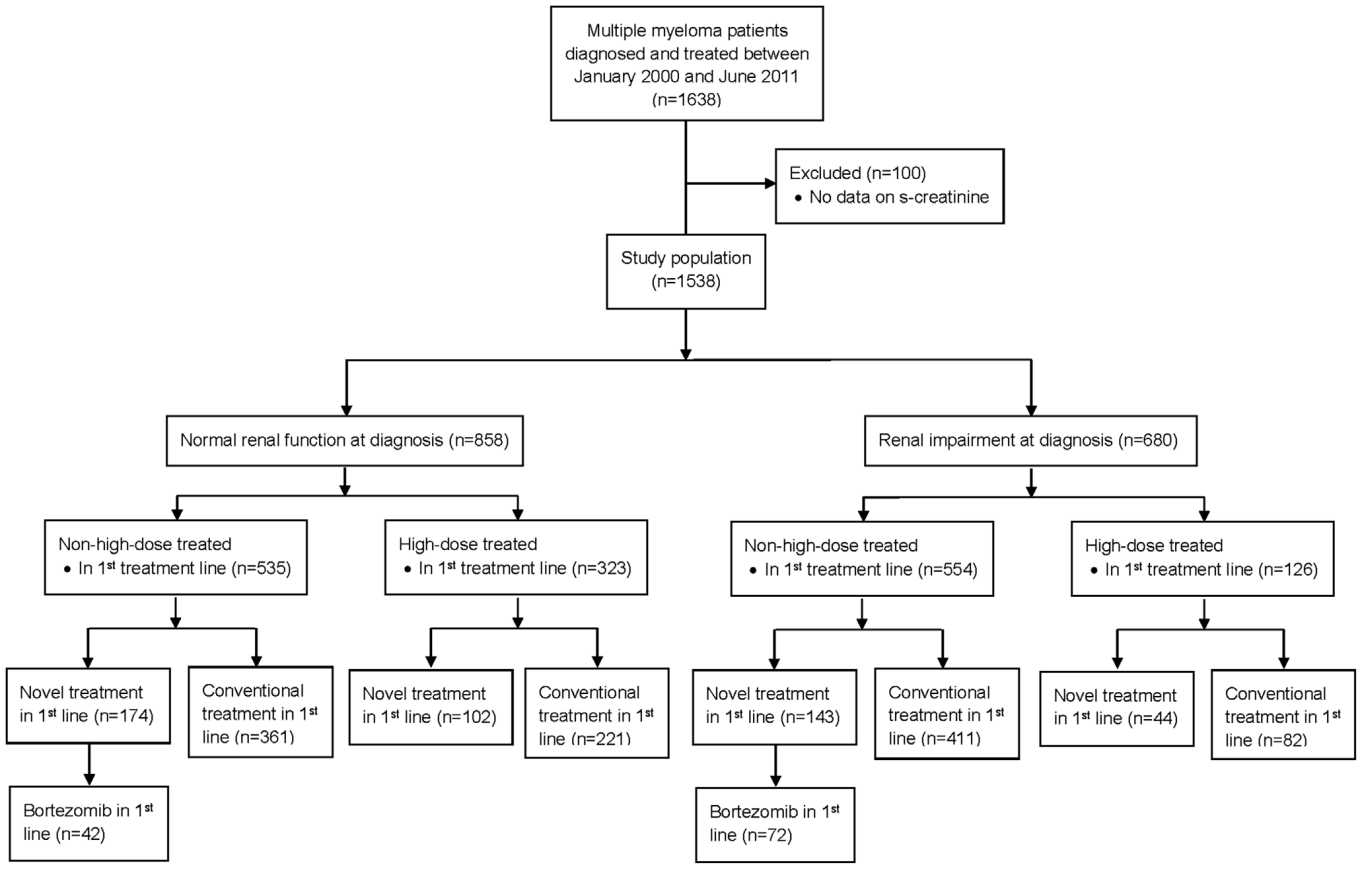
Patients enrolled in this study.

The study population was divided according to Fig. 1.

To evaluate renal response, all MM patients from our hospital were selected. Patients with a GFR <50 ml/min were identified and for these patients all serum creatinine values were gathered from diagnosis until death or end of follow up. The patients were also followed regarding changes in body weight. This cohort was divided into those receiving bortezomib and those receiving other drug combinations (control group). The control group consisted of different treatment regimes: melphalan-based, cyclophosphamide-based, VAD-based (vincristine, adriamycin and prednisone), IMiDs-based and other single drugs or drug combinations.

### Definition of endpoints

Near complete response was defined as an undetectable M-protein in the blood and urine by standard electrophoresis but was not always confirmed by immunofixation since it was not required in clinical practice. Very good partial response (VGPR) was defined as at least 90% reduction of M-protein or a decrease to <200 mg/24 h in the urine M-protein levels. Partial response (PR) was defined as a 50% reduction in M-protein. No response (NR) was defined as less than 50% reduction of M-protein. Progress was considered when M-protein increased by 25% in serum and/or urine, absolute increases of ≥5 g/L in serum, or ≥200 mg/24 hours in urine [19]. Time to next treatment was defined as the time between the start date of the current treatment line and the start date of the next treatment line, death or last follow-up. OS was calculated from the start of the treatment until death or last follow-up.

Renal response was assessed according to the criteria suggested by Ludwig et al and the International Myeloma Group as either a complete response (CRrenal) defined as an improvement of GFR from <50 mL/min at baseline to ≥60 mL/min; a partial response (PRrenal) defined as an improvement of GFR from <15 mL/min at baseline to 30–59 mL/min; a minimal response (MRrenal) defined as an improvement of GFR from <15 mL/min at baseline to 15–29 mL/min or from 15–29 mL/min to 30–59 mL/min; or other, when there was no renal response or the response did not reach a MR renal criteria [20], [21].

### Statistical methods

The Mann-Whitney test for uncorrelated means was performed to investigate possible differences between two independent groups. In order to evaluate the variables in contingency tables, the Fisher's Exact Test was performed. Cox regression analyses were used to identify predictive factors and life table curves were calculated according to Kaplan-Meier and compared using log-rank tests. In addition, descriptive statistics were used to characterize the data. A probability value (*P*) of <0.05 was considered as significant.

## Results

### Patients enrolled in this study

The patients enrolled in this study were divided according to Fig. 1.

The median age at diagnosis was 73 years and 51% of the patients were male. The most common subtype was IgG (53%) followed by IgA (21%), Bence Jones (21%) and other (3%); 63% of the patients had RI stage 3, 21% stage 4 and 17% stage 5. Population characteristics are shown in (Tables 2–3).

**Table 2:** Population characteristics for all patients comparing those with and without renal impairment.

		HDT			Non-HDT		
		Renal impairment			Renal impairment		
		Yes	No	*P*	Yes	No	*P*
**No. of patients**		224	258		456	600	
**Mean age**		58	59	0.76	76	75	0.66
**Type of MM, %**							
	IgG	50.0	59.3		54.8	62.8	
	IgA	25.0	26.0		19.3	21.8	
	BJ	19.2	8.9		21.9	10.2	
	Others	4.9	5.0		2.4	4.7	
	Missing	0.9	0.8		1.5	0.5	
**Laboratory values at diagnosis, mean**							
	Albumin, g/L	32	34	0.006	33	34	0.005
	Hb, g/L	100	114.2	<0.001	102	114	<0.001
	Ca, mmol/L	2.6	2.4	<0.001	2.6	2.4	<0.001
	β2μ, mg/L	9.5	3.3	<0.001	8.1	3.3	<0.001
**Stage of renal function, %**							
	1	0	34.6		0	33.3	
	2	0	65.4		0	66.7	
	3	57.8	0		65.1	0	
	4	22.9	0		18.9	0	
	5	19.3	0		16.0	0	
**Novel drugs, %**							
	Yes	25.6	36.2		30.5	28.0	
	No	74.4	63.8		69.5	72.0	

The HDT patients are those that at some point have received high-dose treatment, irrespective of treatment line and the non-HDT those that have not. Patients classified has having received novel drugs implies that they have received this treatment in one or more treatment lines.

HDT, high-dose treated; non-HDT, non-high-dose treated; MM, multiple myeloma; novel drugs, bortezomib, lenalidomide or thalidomide; IgG, immunoglobulin G; IgA, immunoglobulin A; BJ, Bence Jones; Hb, hemoglobin; Ca, calcium; β2μ, beta-2-mikroglobulin.

**Table 3:** Population characteristics for patients with renal impairment treated with novel agents.

		HDT with renal impairment			Non-HDT with renal impairment		
		Novel treatment			Novel treatment		
		Yes	No	*P*	Yes	No	*P*
**No. of patients**		57	166		135	308	
**Mean age**		59	58	0.62	75	75	0.67
**Type of MM, %**							
	IgG	49.1	50		53.3	55.8	
	IgA	31.6	22.9		17.8	19.8	
	BJ	15.8	20.5		25.2	20.5	
	Other	3.5	5.4		2.2	2.3	
	Missing	0	1.2		1.5	1.6	
**Laboratory values, mean**							
	Albumin, g/L	30	33	0.03	33	32	0.76
	Hb, g/L	98	100	0.46	102	101	0.70
	Ca, mmol/L	2.5	2.6	0.34	2.6	2.6	0.24
	B2M, mg/L	10.2	9.2	0.72	8.3	8.0	0.78
	Creatinine	314	238	0.04	295	217	0.002
**Stage of renal function, %**							
	3	52.6	59.6		60	67.2	
	4	21.1	23.5		18.5	19.2	
	5	26.3	16.9		21.5	13.6	

The HDT patients are those that at some point have received high-dose treatment, irrespective of treatment line and the non-HDT those that have not. Patients classified has having received novel drugs implies that they have received this treatment in one or more treatment lines.

HDT, high-dose treated; non-HDT, non-high-dose treated; MM, multiple myeloma; IgG, immunoglobulin G; IgA, immunoglobulin A; BJ, Bence Jones; Hb, hemoglobin; Ca, calcium; β2μ, beta-2-mikroglobulin.

Of the 1538 patients 556 came from our hospital. Of these 95 patients had a GFR <50 mL/min at diagnosis.

### Overall survival in patients with or without renal impairment

**Figure 2:**
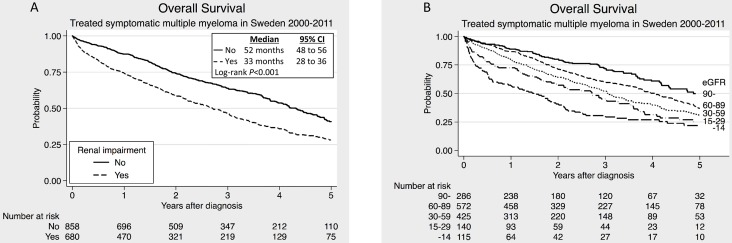
Overall survival in symptomatic multiple myeloma patients. (A) Patients presenting with renal impairment compared to those with normal renal function. (B) Patients divided into five groups according to the chronic kidney disease classification.

Patients with RI at diagnosis had a significantly worse median OS than those without RI (Fig. 2A). Further, there was a clear correlation between the degree of RI and OS (Fig. 2B).

**Figure 3:**
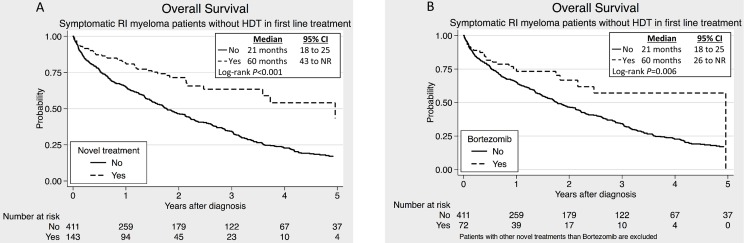
Overall survival for non-high-dose treated (non-HDT) patients with renal impairment. (A) Patients treated with novel agents (bortezomib, thalidomide or lenalidomide) compared to conventional agents in the 1^st^ treatment line. (B) Patients treated with bortezomib compared to conventional agents in the 1^st^ treatment line.

**Figure 4:**
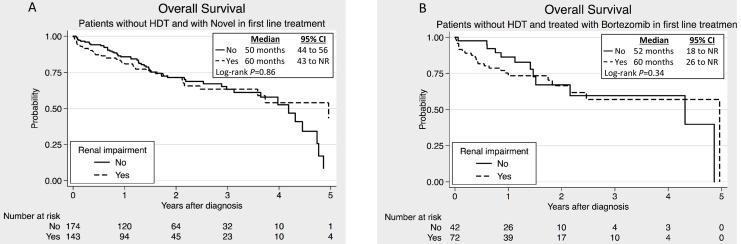
Overall survival in non-high-dose treated (non-HDT) patients with and without renal impairmet treated with novel agents. (A) Patients with renal impairment compared to those without renal impairment after the use of novel agents (bortezomib, lenalidomide or thalidomide) in the 1^st^ treatment line. (B) Patients with renal impairment compared to those without renal impairment after the use of bortezomib in the 1^st^ treatment line.

In the non-HDT group the existence of renal failure correlated with impaired OS (25 versus 37 months, *P*<0.001). Novel drugs in 1^st^ line significantly improved the median OS in this group (Fig. 3A). The difference was still highly significant (*P*<0.001) after correction for age, calcium, hemoglobin and albumin in multivariate analysis. The same improvement was seen when analyzing only the bortezomib treated patients (Fig. 3B). Both the univariate and the multivariate analysis showed that bortezomib treatment was an independent factor affecting OS (Tables S1 and S2). On the other hand, no significant difference in median OS was observed among the patients treated with novel agents in 1^st^ line when comparing the RI patients to those with normal renal function (Fig. 4A). When analyzing only the bortezomib treated patients, the same tendency was seen (Fig. 4B).

On the other hand, in the HDT eligible patients with RI, the median OS was 74 months compared to 91 months for HDT-patients without RI and there was no significant difference in OS between those treated with novel agents and those treated with conventional cytotoxic drugs (*P* = 0.96).

### Novel agents improved response to treatment among the patients with renal impairment

**Figure 5:**
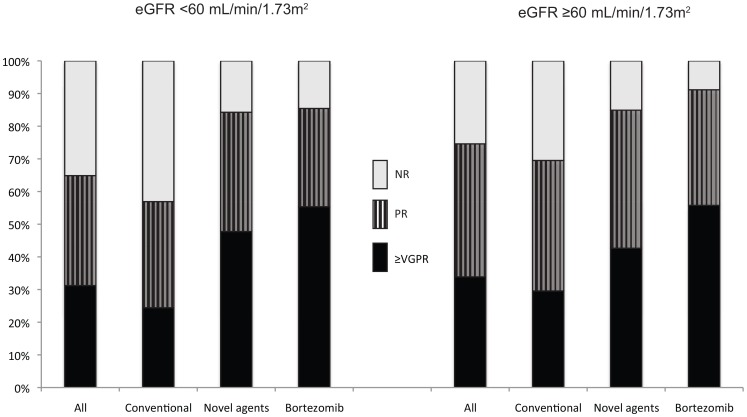
Response distribution after the 1^st^ treatment line in patients with and without renal impairment. Comparing the population treated with novel agents (bortezomib, lenalidomide or thalidomide) to the population treated with conventional agents. (A) Patients with renal impairment. (B) Patients without renal impairment. NR, no response; PR, partial response; ≥VGPR, at least very good partial response.

Non-RI patients had a higher response rate than the RI patients: 74% reached at least PR after 1^st^ line treatment compared to 65%. However, after using novel agent in the 1^st^ treatment line 85% of the RI patients reached at least PR, which can be compared to 56% for the RI patients treated with conventional agents. Of the bortezomib treated patients 85% reached at least PR after the 1^st^ treatment line (Fig. 5).

### Renal response

Of the patients treated with bortezomib in 1^st^ line 11 of 12 (92%) improved their GFR (≥MRrenal) compared to 57 of 83 patients (69%) in the control group (*P* = 0.049). Four of the 12 (33%) bortezomib treated patients and 38 of 83 patients (46%) in the control group reached CRrenal after 1^st^ treatment line.

### Novel agents and HDT prolonged time to next treatment

**Figure 6:**
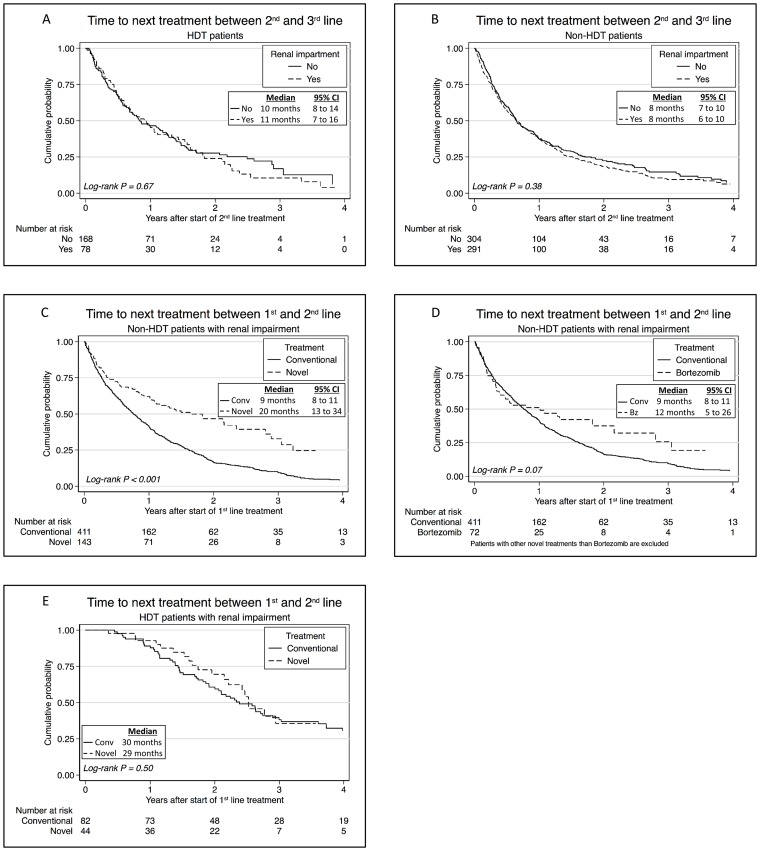
Time to next treatment in patients with and without renal impairment at diagnosis. (A) In high-dose treated (HDT) patients with and without renal impairment after 2^nd^ line of therapy. (B) In non-HDT patients with and without renal impairment after 2^nd^ line of therapy. (C) Non-HDT patients with renal impairment treated with novel agents (bortezomib, lenalidomide or thalidomide) in the 1^st^ line compared to those treated with conventional agents (Conv.). (D) Non-HDT-patients with renal impairment treated with bortezomib (Bz) in the 1^st^ line compared to those treated with conventional agents. (E) HDT-patients with renal impairment treated with novel agents (bortezomib, lenalidomide or thalidomide) in the 1^st^ line compared to to those treated with conventional agents.

RI implied a shorter median TTNT after 1^st^ line (13 versus 20 months, *P*<0.001). However, TTNT between 2^nd^ and 3^rd^ treatment lines was not affected by renal function at diagnosis, neither among the HDT-patients nor among the non-HDT-patients (Fig. 6A–B).

Additionally, the non-HDT patients with RI had a median TTNT after 1^st^ line of 11 months, compared to 14.5 months for patients without RI (*P*<0.001). Novel drugs prolonged the TTNT after 1^st^ line in these patients (Fig. 6C) and bortezomib treatment lead to a significant improvement of TTNT after adjusting for age, calcium, hemoglobin and albumin (*P* = 0.02) (Fig. 6D).

On the contrary, the HDT population with RI had a median TTNT after 1^st^ line of 30 months compared to patients without RI who had a median TTNT of 35 months (*P* = 0.159) and novel agents in 1^st^ line did not affect median TTNT in this population (Fig. 6E).

## Discussion

Our main finding is that novel agents, especially bortezomib, are associated with survival benefits in MM patients with RI in a real life setting. By using novel agents the OS among RI patients not eligible for HDT was significantly improved from a median 21 to 60 months. Bortezomib-based regimens also improved the median OS to 60 months and was an independent factor to enhance OS in non-HDT patients with RI (*P* = 0.02). Moreover, the difference in survival between those with and without RI vanished with the use of novel agents. The median TTNT between the 1^st^ and 2^nd^ treatment lines in non-HDT patients with RI was more than doubled by the use of novel agents. However, the same effect on TTNT was not seen in the HDT group.

Renal impairment is a frequent complication of MM and has been associated with poor outcome. The present study investigated the impact of RI on survival and myeloma response in a large cohort of MM patients treated in a real life setting; all consecutive cases were obtained from the Swedish Cancer Registry. The data in this study derive from several different hospitals, not only referral centers. Therefore, we believe that our population represents the full spectrum of different MM patients and we consider the study representative for the whole Swedish MM population.

One reason for the improved OS in RI patients with novel agents is a better disease control [22]. We found a clear improvement of responses when using novel agents compared to the use of conventional agents. In line with the study by Dimopoulos et al [23] we have observed that among RI patients treated with novel agents in the 1^st^ treatment line 85% obtained more than PR. This was significantly better than for those treated with conventional drugs (66%). Interestingly, more than half of the patients with RI treated with bortezomib-based regimens reached at least VGPR (55%) even though the amount of patients reaching at least PR was the same (85%).

We found that renal response occurred more frequently in patients treated with bortezomib compared to conventional agents and this result corresponds with earlier studies [24], [25]. Renal response is correlated to improved myeloma response [25] and improved OS [26]. When renal response coincides with myeloma response the outcome is improved compared to when renal response and myeloma response occur separately [26]. However, renal response can also occur in patients not experiencing a myeloma response [24], [25].

Since this is a retrospective study with data from 15 different hospitals it is influenced by center specific clinical routines. This includes measurement of response, which was mostly done monthly but sometimes every 6–8 weeks. These variations might affect time to progression and progression free survival. However, treatments were started when the physician considered the relapse to be treatment demanding and this is why we consider data on TTNT to be more reliable than time to progression and progression free survival. Since it is more likely that patients treated at university clinics could be included in clinical trials than those treated at local hospitals, it is also impossible to avoid some degree of selection bias in the choice of treatment. One weakness of this study is the lack of information about concomitant diseases, thus making it impossible to correct for comorbidity. However, the results were stratified according to age, sex, type of MM and important laboratory values at diagnosis in an attempt to cover the majority of possible differences between the groups.

Nevertheless, our results concur with earlier studies. A subgroup analysis of patients with RI enrolled in the VISTA study (bortezomib plus melphalan and prednisone versus melphalan and prednisone to previously untreated patients) reports a higher myeloma response rate as well as a longer time to progression and a better OS in the bortezomib plus melphalan and prednisone group [24]. A recent study reports that even though the incidence and severity of RI at diagnosis have been unchanged during the last 20 years the survival among these patients has improved significantly since the introduction of novel agents in 2000. The survival has improved even more after 2005 when the use of bortezomib increased. This indicates an advantage of bortezomib-based therapy, even though the correlation could not be verified [22]. A study analyzing the effect of novel agents on newly diagnosed MM patients with RI found that RI was not independently associated with inferior survival [27]. An earlier study of MM patients presenting with RI (the majority of which had received earlier MM treatment) observed an overall response rate of 80% in previously untreated patients and 58% in relapsed/refractory patients after bortezomib treatment [28].

In conclusion, the use of novel agents such as IMiDs and most importantly bortezomib, improves OS in MM patients with RI leading to the same median OS regardless the existence of RI. We therefore conclude that novel agents can overcome the negative impact of RI and improve the outcome of MM treatment.

## Supporting Information

Table S1 **Univariate analysis of factors affecting overall survival in multiple myeloma patients with renal impairment.** Both HDT and non-HDT patients are included.
(DOCX)

Table S2 **Multivariate analysis of factors affecting overall survival in non-HDT patients treated with bortezomib in 1^st^ treatment line.**
(DOCX)
