# Supplementary material for: The Use of Novel Drugs Can Effectively Improve Response, Delay Relapse and Enhance Overall Survival in Multiple Myeloma Patients with Renal Impairment
Source: PLoS One. 2014 Jul 8;9(7):e101819. doi: 10.1371/journal.pone.0101819 (PMC4086950; doi:10.1371/journal.pone.0101819)
Supplement: Table S1 — Univariate analysis of factors affecting overall survival in multiple myeloma patients with renal impairment. Both HDT and non-HDT patients are included. (DOCX) [file pone.0101819.s001.docx]

# Table S1

| **Covariate** | | **No. of patients** | **Hazard ratio** | **95% Confidence Interval** | ***P*** |
| --- | --- | --- | --- | --- | --- |
| **Sex** |  |  |  |  |  |
|  | Male | 348 | 1.0 |  |  |
|  | Female | 332 | 1.04 | 0.86-1.26 | 0.66 |
| **Myeloma type** |  |  |  |  |  |
|  | IgG | 361 | 1.0 |  |  |
|  | IgA | 144 | 1.31 | 1.03-1.66 | 0.026 |
|  | BJ | 143 | 0.97 | 0.76-1.24 | 0.80 |
|  | Other | 23 | 0.73 | 0.41-1.31 | 0.29 |
| **Skeleton destructions** |  |  |  |  |  |
|  | 0 | 216 | 1.0 |  |  |
|  | 1 | 54 | 0.93 | 0.65-1.35 | 0.72 |
|  | >1 | 376 | 0.95 | 0.77-1.17 | 0.61 |
| **ISS-stage** |  |  |  |  |  |
|  | I | 35 | 1.0 |  |  |
|  | II | 161 | 2.00 | 1.00-3.98 | 0.049 |
|  | III | 220 | 3.14 | 1.58-6.16 | 0.001 |
| **Age** |  | 680 | 1.05 | 1.04-1.06 | <0.001 |
| **Age (per 10 year)** |  | 680 | 1.59 | 1.43-1.76 | <0.001 |
| **Albumin** |  | 662 | 0.97 | 0.96-0.98 | <0.001 |
| **Albumin (per 10 units)** |  | 662 | 0.74 | 0.64-0.85 | <0.001 |
| **Hemoglobin** |  | 670 | 0.99 | 0.99-1.00 | 0.002 |
| **Hemoglobin (per 10 units)** |  | 670 | 0.92 | 0.87-0.97 | 0.002 |
| **Calcium** |  | 637 | 1.36 | 1.11-1.66 | 0.004 |
| **Beta-2-mikroglobulin** |  | 416 | 1.03 | 1.02-1.04 | <0.001 |
| **HDT in 1^st^ line** |  |  |  |  |  |
|  | No | 574 | 1.0 |  |  |
|  | Yes | 106 | 0.34 | 0.25-0.47 | <0.001 |
| **Novel treatment in 1^st^ line** |  |  |  |  |  |
|  | No | 493 | 1.0 |  |  |
|  | Yes | 187 | 0.49 | 0.37-0.65 | <0.001 |
| **Bortezomib treatment in 1^st^ line** |  |  |  |  |  |
|  | No | 570 | 1.0 |  |  |
|  | Yes | 110 | 0.59 | 0.41-0.83 | 0.003 |

IgG, immunoglobulin G; IgA, immunoglobulin A; BJ, Bence Jones; HDT, high-dose treated (melphalan 200 mg/m^2^); novel treatment, bortezomib, lenalidomide or thalidomide.
