# Supplementary material for: The Use of Novel Drugs Can Effectively Improve Response, Delay Relapse and Enhance Overall Survival in Multiple Myeloma Patients with Renal Impairment
Source: PLoS One. 2014 Jul 8;9(7):e101819. doi: 10.1371/journal.pone.0101819 (PMC4086950; doi:10.1371/journal.pone.0101819)
Supplement: Table S2 — Multivariate analysis of factors affecting overall survival in non-HDT patients treated with bortezomib in 1st treatment line. (DOCX) [file pone.0101819.s002.docx]

# Table S2

| **Risk factors** | | **RR** | **95% Confidence Interval** | ***P*** |
| --- | --- | --- | --- | --- |
| **Sex** |  |  |  |  |
|  | Male | 1.0 |  |  |
|  | Female | 0.95 | 0.77-1.18 | 0.68 |
| **Age** |  | 1.04 | 1.02-1.06 | <0.001 |
| **Myeloma type** |  | 1.01 | 0.91-1.12 | 0.79 |
| **Kappa/Lambda** |  | 0.94 | 0.75-1.18 | 0.58 |
| **Skeleton destructions** |  | 0.99 | 0.87-1.11 | 0.83 |
| **Hemoglobin** |  | 0.99 | 0.99-1.00 | 0.02 |
| **Albumin** |  | 0.98 | 0.96-0.99 | 0.006 |
| **Calcium** |  | 1.75 | 1.36-2.24 | <0.001 |
| **Beta-2-mikroglobulin** |  | 1.03 | 1.02-1.04 | <0.001 |
| **Treatment** |  |  |  |  |
|  | Conventional | 1.0 |  |  |
|  | Bortezomib | 0.85 | 0.74-0.97 | 0.02 |
